# Selection and Amplification of Fungicide Resistance in *Aspergillus fumigatus* in Relation to DMI Fungicide Use in Agronomic Settings: Hotspots versus Coldspots

**DOI:** 10.3390/microorganisms9122439

**Published:** 2021-11-26

**Authors:** Kevin J. Doughty, Helge Sierotzki, Martin Semar, Andreas Goertz

**Affiliations:** 1 Bayer AG, Alfred Nobel Strasse 50, 40789 Monheim-am-Rhein, Germany; andreas.goertz@bayer.com; 2 Syngenta Crop Protection, Schaffhauserstrasse 101, 4332 Stein, Switzerland; helge.sierotzki@syngenta.com; 3 BASF SE, Speyerer Strasse 2, 67117 Limburgerhof, Germany; martin.semar@basf.com

**Keywords:** *Aspergillus fumigatus*, DMI fungicides, azoles, resistance, agriculture, mitigation

## Abstract

*Aspergillus fumigatus* is a ubiquitous saprophytic fungus. Inhalation of *A. fumigatus* spores can lead to Invasive Aspergillosis (IA) in people with weakened immune systems. The use of triazole antifungals with the demethylation inhibitor (DMI) mode of action to treat IA is being hampered by the spread of DMI-resistant “ARAf” (azole-resistant *Aspergillus fumigatus*) genotypes. DMIs are also used in the environment, for example, as fungicides to protect yield and quality in agronomic settings, which may lead to exposure of *A. fumigatus* to DMI residues. An agronomic setting can be a “hotspot” for ARAf if it provides a suitable substrate and favourable conditions for the growth of *A. fumigatus* in the presence of DMI fungicides at concentrations capable of selecting ARAf genotypes at the expense of the susceptible wild-type, followed by the release of predominantly resistant spores. Agronomic settings that do not provide these conditions are considered “coldspots". Identifying and mitigating hotspots will be key to securing the agronomic use of DMIs without compromising their use in medicine. We provide a review of studies of the prevalence of ARAf in various agronomic settings and discuss the mitigation options for confirmed hotspots, particularly those relating to the management of crop waste.

## 1. Introduction

### 1.1. Aspergillus fumigatus

*Aspergillus fumigatus* is a ubiquitous saprophytic ascomycete fungus typically found in nature growing on decaying vegetation on and in soil [1,2,3]. It is thermotolerant, with a broad temperature range for growth (12–65 °C, optimum growth temperature of ~35 °C), and it benefits from high humidity (85–100%), characteristics that allow it to thrive in plant waste heaps [4,5,6].

*A. fumigatus* is a widespread constituent of the air microflora. Prolific release of conidia [1,7] from natural sources or others associated with human activity (e.g., composting, building work) can lead to high spore counts in aerosols, reaching up to 10^6^ colony-forming units (CFUs) per m^3^ [8], depending on the location, context, and season. People can inhale *A. fumigatus* spores from the air in both the indoor and outdoor environments [2,4].

*A. fumigatus* is also the most important of several *Aspergillus* species capable of opportunistic infection of humans and many species of domesticated animal [4,9,10]: its clinical effects range from allergic reactions to chronic pulmonary Aspergillosis (CPA) and acute invasive Aspergillosis (IA) in immunocompromised patients [11,12,13].

### 1.2. Demethylation Inhibitor (DMI) Antifungals in the Clinic and Fungicides in the Environment

The most important DMI antifungals used in medicine and DMI fungicides used in the outside environment (often referred to generically as “azoles” in the scientific literature) belong to the triazole and imidazole groups, which share the ability to inhibit sterol 14-alpha-demethylase, a member of the P450 family encoded by the *cyp51* gene. Sterol demethylation is intrinsic to the biosynthesis of ergosterol, an essential component of fungal membranes, the lack of which causes a loss of functionality, followed by fungistasis [13,14,15].

Since their first introduction in the 1970s, DMI fungicides have been used for various purposes in the outside environment: as crop protection agents in agriculture and horticulture; as material protectants (e.g., in timber treatment, paints), and for veterinary treatments [4,12,16]. In agriculture, DMI fungicides continue to be indispensable crop protectants for foliar application and seed treatment [17,18], a position they have maintained over several decades because their high-level, broad-spectrum efficacy has been largely resilient towards loss of efficacy against target pathogens: they show partial “shifting” resistance only, in contrast to the full resistance that has developed quite rapidly to other modes of action [12,19].

Many triazoles and imidazoles are active against *A. fumigatus* [20,21], hence the introduction of medical triazole therapies to combat CPA and IA in the late 1980s [6,10]. Triazoles are currently the first-line choice of medical treatment for patients suffering from these conditions. Therapeutic alternatives to triazoles exist (echinocandins and polyenes), but unlike triazoles, they cannot be applied orally (resulting in higher cost) and are less favourable in terms of efficacy and side effects. Thus, they are generally administered in combination with triazoles or are positioned as salvage treatments if triazole therapy fails [9,10,12,13,15].

### 1.3. Resistance to DMI Fungicides in A. fumigatus

Resistance to DMIs in *A. fumigatus* was first observed in the clinical setting in the late 1990s, followed by a rise in incidence over the last two decades [15,22]. The initial findings were followed by the recovery of resistant isolates from patients in many countries [9,23]. Resistant isolates hinder triazole therapy, and the infection with DMI-resistant *A. fumigatus* (ARAf) is associated with significantly higher patient mortality [9,10,12,13,15,24,25,26,27].

Triazole resistance can be acquired during therapy, particularly in patients undergoing long-term DMI treatment [10,15,28]. ARAf isolates recovered from patients typically carry resistance mechanisms based on point mutations at various positions in the *cyp51A* gene (which decrease the affinity of DMIs for the target protein), altered efflux pump activity, or increased *cyp51A* copy number [11,15] as well as other mechanisms [9,12,13,14]. However, a recent finding indicates that a mutation in the *cyp51B* gene can also confer DMI resistance [29].

Strong evidence for the emergence of resistance within patients during DMI therapy has been provided by applying microsatellite typing to isolates recovered serially from infected individuals [10,11,30]. In-patient emergence of triazole resistance is probably associated with pulmonary cavities, within which the fungus can sporulate asexually from an aspergilloma, providing an opportunity for resistant variants to emerge that are then capable of being selected by the medical DMIs [6,11,12,23].

The recovery of resistant isolates from triazole-naïve IA patients in the late 1990s indicated that the external environment presents an additional route for ARAf infection [11,15,20,25,31,32]. “Environmental” ARAf strains typically combine the insertion of tandem repeats (TRs) in the promoter region of the *cyp51A* target gene (which up-regulate the expression of *cyp51A*) together with single or multiple point mutations in the coding sequence of the gene [9,12,15,33]. The most ubiquitous common mutants recovered from patients that are generally held to be of environmental origin are TR_34_/L98H, TR_46_/Y121F/T289A, and TR_34_/L98H/S297T/F495I [9,10,34,35,36]. TR_34_/L98H, which combines a 34 base-pair tandem repeat with a leucine-to-histidine substitution at position 98 in the amino acid coding sequence, was first recovered from a Dutch patient in 1999 [37,38]. It was later found in a sample taken from an Italian patient in 1998 [39], and it has since been detected in clinical settings (e.g., hospital corridors, intensive care units) and recovered from patients and environmental samples from around the globe [15,25,33,40]. A second widespread “environmental” mutant, TR_46_/Y121F/T289A, was first recovered from a Dutch patient in 2009 [9,10,41,42] and later found in a sample from a US patient taken in 2008 [43]. It too has been detected widely in the environment [44], albeit less frequently than TR_34_/L98H [45]. Further TR-associated mutants, such as a TR_53_ mutant without substitutions in the *cyp51A* gene, have also been detected [9,46].

While there is some evidence for in-patient emergence of TRs [11,47,48] and nosocomial transfer (i.e., within the clinic) of ARAf isolates [49], the environment is likely to be a relevant source of IA patient contamination with resistant isolates [6]. Several studies have shown correspondence between specific ARAf genotypes isolated from patients and the general or their local environments [15,50,51,52]. ARAf carrying the TR_34_ mutation has also been detected in hospital air and dust samples [53,54,55], as well as hospital gardens, suggesting that the immediate hospital surroundings can contribute to the clinical spore load [53].

A number of DMI fungicides used in the outside environment are intrinsically active against *A. fumigatus* [20,21,56,57]. Cross-resistance in ARAf isolates to these DMIs and the different triazoles used in medicine is common, but not always the case, depending on the genetic configuration of the ARAf genotype [20,52,57]. Unlike other Aspergillus species such as *Aspergillus flavus* and *Aspergillus niger*, *A. fumigatus* is not able to infect living plant tissues, so it is not a crop pathogen and therefore not a target of fungicide applications in agriculture [4]. However, “collateral” exposure of the fungus is possible in environmental settings. For example, DMI fungicides applied to crops start to dissipate after treatment; however, their presence in the treated field can also extend beyond the cropping cycle in the form of steadily declining residues in the soil [58] and on non-harvested parts of the crop, including vegetative substrates capable of colonization by *A. fumigatus*. If DMI residues are present in these substrates at concentrations sufficient to inhibit the growth of the wild-type portion of the *A. fumigatus* population, but not that of resistant isolates, then this convergence may lead to the selection of ARAf. Snelders et al. [20] identified a group of five agricultural DMI fungicides with structural similarity (and cross-resistance) to medical triazoles and pointed out that the timing of market introduction of these compounds immediately preceded the estimated date of origin of TR_34_/L98H. Toda et al. [59] also related the first recovery of a TR_34_ mutant from a patient in the United States in 2016 to the preceding decade of intensification of national agricultural DMI use. A further indication of the potential participation of agronomic settings in selection for resistance derives from the characterization of ARAf isolates—deriving from clinical, environmental and plant material sources—that are also resistant to other modes of action which are used in agriculture but not in medicine, such as the quinone outside inhibitor (QoI), methyl benzimidazole carbamate (MBC), and succinate dehydrogenase inhibitor (SDHI) fungicide classes [60,61,62].

### 1.4. Emergence of Resistance in A. fumigatus during Exposure to Agricultural DMIs

Environmental saprophytic fungi such as *A. fumigatus* are often phenotypically and genotypically plastic, allowing them to respond to stressful stimuli (environmental factors, nutrient availability, competitive stress, toxins) via stable genetic or epigenetic changes that are then capable of selection [10].

Laboratory studies have demonstrated the emergence of *cyp51A* gene mutations during exposure to agricultural DMIs in culture media or soil, with mutants showing increased MICs (Minimum Inhibitory Concentrations) and either pan-DMI resistance or resistance to specific medical DMIs [20,63,64,65,66,67,68,69]. For example, Snelders et al. [20] showed the emergence, during in vitro exposure to agricultural DMIs, of *cyp51A* substitutions that are also known to emerge within patients during therapy. They did not detect the emergence of TR_34_/L98H, although a tripling of the 34 base-pair tandem repeat occurred in an isolate already possessing the TR_34_/L98H mutation. In contrast, Ren et al. [64] reported the recovery of the resistant genotype TR_46_/Y121F/T289A after repeated subculturing of an initially wild-type strain on medium supplemented with agricultural DMIs, and Cao et al. [67] recovered TR_34_/L98H and TR_34_/L98H/S297T/F495I mutants from tomato leaves and soils treated with the agricultural DMI tebuconazole.

The emergence of mutants in these studies (and in the environment) is assumed to be the result of the spontaneous generation of genetic changes followed by selection under DMI pressure. DMIs have not been shown to be mutagenic: mass production of conidia from fungal colonies provides ample opportunity for spontaneous mutations to occur during the myriad mitotic events leading to the formation of spore masses [6,70]. Evidence for the role of asexual sporulation in the emergence of resistant mutants during exposure to agricultural DMIs in vitro was provided by Zhang et al. [71], who later showed the emergence of a TR_34_^3^/L98H isolate following asexual reproduction of a TR_34_/L98H ancestor in the presence of voriconazole [72].

Alterations in the promotor region (of which tandem repeats are an example) and *cyp51A* single nucleotide polymorphisms typical of environmental ARAf isolates have generally been found to emerge separately in DMI-resistant isolates of plant pathogens directly targeted with agricultural DMIs [9]. However, a combination of these resistance mechanisms has recently been found in the environment in isolates of the plant pathogens *Pyrenopeziza brassicae* and *Pseudocercospora fijiensis* showing decreased sensitivity to DMIs [73,74].

The mutations leading to DMI resistance in ARAf do not generally impose a significant fitness cost, allowing them to survive and spread in the absence of DMIs [11,23,51]. Verweij et al. [70] raise the possibility that tandem repeats in the promoter region may provide a compensatory mechanism for metabolically costly point mutations in the *cyp51A* gene.

### 1.5. Population Dynamics of Azole-Resistant A. fumigatus in the Environment

DMI resistance is present in environmental populations of *A. fumigatus* around the globe [13,45]. There is evidence that TR-associated resistance has arisen rarely in the environment but has subsequently spread widely, acquiring various point mutations in the meanwhile [12,75]. For example, genomic analysis of both clinical and environmental TR_34_/L98H and TR_46_/Y121F/T289A isolates from different geographical locations [35], across India [76] and from across the UK and Ireland [51] showed that the TR-associated DMI-resistant genotypes show relatively low genetic diversity, evidence that frequent emergence of these mutations is unlikely. The less common TR_53_ mutation first reported from the clinical setting in the Netherlands in 2009 has since been detected in the environment in Europe as well as in Colombia [15,77].

*A. fumigatus* conidia can be dispersed over long distances in the air, and it is conceivable that the global distribution of TR_34_/L98H and TR_46_/Y121F/T289A is the result of conidial spread, as shown by the detection of identical clones in different European countries [60] and by the isolation of identical TR_34_/L98H and TR_46_/Y121F/T289A clones from the environment and clinical settings worldwide [35]. However, the transfer of ARAf isolates on plant material such as flower bulbs has been demonstrated [53], and the long-distance spread of resistant isolates by birds is also considered a possibility [78].

### 1.6. Purpose

The following section provides a survey of the detection of ARAf genotypes in various agronomic settings. Based on this overview, we discuss whether it is possible to characterize individual agronomic settings as so-called “hotspots” (settings capable of providing suitable conditions for the “amplification” and spread of resistant genotypes), or conversely as “coldspots” (settings that do not provide these conditions). Allocating agronomic settings into these categories will be a necessary step towards identifying the situations in which the use of agricultural DMIs might trigger the need for mitigation measures to limit the amplification and spread of ARAf genotypes.

This review does not consider the use of DMIs in other, non-agronomic environmental contexts, such as material preservation, which can bring *A. fumigatus* into contact with residues of DMI fungicides, and which has also been studied in terms of the amplification of resistant isolates, for example in sawmills [16,79]. These uses may, to some extent, converge with agronomic uses at the point of waste management [56]. The implications of DMI use in veterinary medicine [80] are not covered here.

## 2. Characteristics of Agronomic Hotspots for the Amplification and Spread of ARAf

An agronomic hotspot is an agricultural or horticultural setting that fulfils the following conditions: it provides a substrate capable of supporting the growth, reproduction and dispersal of a population of *A. fumigatus*: for selection of resistant genotypes to occur, the prevailing environmental conditions must also allow *A. fumigatus* to grow and complete its life cycle to the point of spore production, and the substrate must contain DMI residues at concentrations capable of selecting ARAf isolates from within a mixed population of susceptible and resistant genotypes [5]. The greater the population size, the greater the probability that mutations conferring DMI resistance emerge that are then capable of selection [11].

Within a hotspot, the selective action of DMI residues results in the resistant proportion of the population being “amplified,” i.e., its ability to develop and release propagules into the environment is favoured at the expense of the DMI-susceptible portion [12,56]. This can increase the proportion of ARAf spores in the air spora over “background” levels (Table 1). Mass release of conidia is considered essential to the definition of an ARAf hotspot because airborne conidia are the predominant route of infection of patients by *A. fumigatus* deriving from the environment [3,4,15].

## 3. Review of the Prevalence of *A. fumigatus*/ARAf in Various Agronomic Settings

### 3.1. Background Data

Supplementary Tables S1–S4 provide a global overview of studies reporting the isolation (or absence) of *A. fumigatus* and ARAf genotypes from various matrices (soil, plant materials, etc.) in relation to the use of DMI fungicides in different agronomic settings. Coverage is restricted to studies that specify which agronomic setting(s) were sampled and those involving the detection of *A. fumigatus* sensu stricto. The tables follow the designation of resistance resulting from the respective authors’ interpretation of MIC values. No attempt is made to report the frequency of ARAf isolates in the different agronomic settings, as this depends strongly on sampling and isolation methodology.

### 3.2. Comparison of Urban and Agronomic Settings

It is clear from the literature that the prevalence of *A. fumigatus* (and ARAf) varies significantly within and between different settings, with often contradictory findings. An area that illustrates this is the comparison of detection frequencies in urban and agronomic settings. Astvad et al. [81] found no ARAf genotypes in 239 soil samples taken from either agricultural or urban settings in Denmark. Among several studies performed in the United Kingdom, Bromley et al. [82] found no ARAf in uncultivated soil from urban locations but were able to isolate ARAf resistant to itraconazole (1.7% of samples) from agricultural soil taken from fields in which cereals had been grown with a history of DMI treatment. In contrast, Tsitsopoulou et al. [83] found more ARAf in public urban (8.4% of samples) than in agricultural soils (5.2%), but the difference was not statistically significant. Sewell et al. [35] found the highest incidence of ARAf, with the greatest genotypic diversity, in soils from the urban environment of central London, rather than in samples from farmland, woodland, and pasture. A study of soil samples taken in five countries in South America and Africa [84] showed that ARAf was more prevalent in samples from urban sites than in those from agricultural land: mutants including TR_34_/L98H and TR_46_/Y121F/T289A were found in DMI-contaminated agricultural soils (in Peru), but also in urban flower bed soils lacking DMI residues (in Mexico and Paraguay). Tangwattanachuleeporn et al. [85] did not recover ARAf isolates from soil sampled in metropolitan areas of Thailand, but they found resistant isolates in only ca. 3% of soil samples from agronomic settings. In contrast, Duong et al. [40] found that the likelihood of detection of itraconazole-resistant ARAf in air samples taken in Vietnam was greatest in municipal areas. In their review of a bloc of 52 studies investigating the occurrence of ARAf in the environment, Burks et al. [45] found that more resistant isolates were collected in total from developed environments than from agricultural settings, despite the latter being sampled more often. The pattern emerging from these studies is that ARAf is generally more prevalent in urban than in agricultural areas.

Contradictions among studies may in part be due to the timing of sampling, at least in temperate climates. The prevalence of *A. fumigatus* tends to be seasonal in nature in Northern Europe, tending to increase in winter in association with the general onset of decay of organic matter [1,3]. In Germany, Barber et al. [86] found *A. fumigatus* to be most abundant in both organic and DMI-treated agricultural soils in November, after harvest. In contrast, there was no seasonal trend in *A. fumigatus* spore counts in air samples from an urban environment in the UK [87]. Sampling is further confounded by the significant variation in the size of the *A. fumigatus* population in the soil, both between fields and within the same field, as well as by the dependency of findings on sampling depth [86].

### 3.3. ARAf in Flower Bulb Cultivation Settings

In flower bulb cultivation (Supplementary Table S1), DMIs are applied directly to the bulbs via pre-planting dipping or as foliar sprays to plants in the field, mainly to protect against diseases caused by *Fusarium oxysporum* and *Botrytis* spp. [5,12]. This agronomic setting has been investigated particularly intensively in the Netherlands, where much scientific knowledge has been generated, indicating that DMI use in bulb production is a potential ARAf hotspot, particularly in connection with plant waste piles. Leaf material, reject bulbs, and outer scales are removed from harvested bulbs before the latter are sold (or replanted), and some flower bulb growers stockpile this waste in field edge heaps before composting. These waste heaps can provide a conducive substrate for *A. fumigatus*, and the DMI residues they contain are capable of amplifying ARAf genotypes [5,52]. Compost created from DMI-untreated flower bulb waste was found to be almost free of resistant isolates, while resistant isolates were predominant in composting materials with a known history of DMI treatment [38,52]. In some studies comparing various matrices (including agricultural soils), flower bulb waste has been found to contain more *A. fumigatus* CFUs and greater ARAf genotype diversity than other sources [5]. Isolates detected in flower bulb waste in the Netherlands showed similar genetics of resistance to those recovered from patients in Dutch hospitals [38,52].

### 3.4. ARAf in Arable Crop Settings

Among the arable crops (Supplementary Table S2), cereals represent the most intensively researched setting with regard to the incidence of ARAf. Sewell et al. [35] found lower rates of *A. fumigatus* detection in wheat farm soils than in municipal park soils, and no ARAf was found among the strains isolated from cereal fields. Although Prigitano et al. [88] found *A. fumigatus* in all cereal soils sampled in Italy, none of the isolates showed itraconazole resistance. Rocchi et al. [89] found ARAf in one of 18 samples of soil and leaves from a cereal field in France. Tsitsopoulou et al. [83] found low frequencies of ARAf in UK cereal soils, whereas Bromley et al. [82] found ARAf in two of four UK wheat fields sampled. Trovato et al. [90] found ARAf in soil and leaf samples from cereal fields in one of two areas sampled in Sicily, but *A. fumigatus* was absent from cereals fields in the second area. Fraaije et al. [60] sampled soils from the Broadbalk long-term winter wheat study in the UK, which allowed direct comparison of untreated plots with others that had received annual DMI treatments since the time of first market introduction. Itraconazole resistance was found in less than one per cent of isolates from the Broadbalk soils, and pan-azole resistance was not detected (i.e., an absence of TR_34_ and TR_46_ mutants). These authors also found a maximum of 5.1% detection of pan-azole resistant isolates in DMI-treated soils from the neighbouring Woburn farm and a low frequency of detection (0.5%) of pan-azole resistance in commercial wheat soils from elsewhere in the UK, France, and Germany, with ARAf being absent from a wheat field soil sampled in Hungary. Barber et al. [86] sampled cereal soils in an area of Germany associated with intense DMI use; they found a low overall incidence for ARAf, and the incidence did not differ between the DMI-treated and organic cereal farms sampled. The most common ARAf isolate in 2016 and 2017 was TR_34_/L98H, whereas in 2018, only two resistant isolates were detected, both of which had wild-type *cyp51A* loci. Suppression of the general *A. fumigatus* population was observed following DMI application that was absent from the organic field soil; however, this effect was noted in only one of two years of sampling. The authors further noted that the application of DMIs in the field was followed by an increase in the proportion of sampled isolates capable of growing on tebuconazole- or difenoconazole-amended agar, but this change was transient. Prigitano et al. [91] recovered TR_34_/L98H from a cereal field in Sicily that had not been treated with DMIs.

Schoustra et al. [5] did not detect *A. fumigatus* on cereal grain (from either conventional or organic cropping), and cereal straw does not appear to be a suitable substrate for the growth of *A. fumigatus* [4,5]. Fraaije et al. [60] showed that the incorporation of cereal straw into soil did not increase the (low) rate of recovery of pan-azole resistant isolates.

Studies in France have shown a lack of correspondence between isolates recovered from patients and those detected in their adjacent arable crop environment. Rocchi et al. [89] found a TR_34_ isolate in a barley field adjacent to a patient’s home but showed that the isolate was not genetically linked to the TR_34_ isolate recovered from the patient. A subsequent investigation [50] revealed the immediate residential and (vegetable) garden environment of a second patient as the most likely source of the TR_46_/Y121F/T289A isolate to which he succumbed; soil samples from neighbouring maize and wheat fields did not harbour ARAf, and a neighbouring barley field again harboured a TR_34_/L98H isolate.

Taken together, these findings indicate that cereals soils do not generally support large *A. fumigatus* populations, nor do they harbour significant proportions of ARAf, suggesting that the cereals setting is a cold spot—a conclusion also reached by Fraaije et al. [60] and Barber et al. [86]. The proportions of ARAf detected in populations recovered from cereal soils may reflect background levels; for example, Fraaije et al. [60] found the low levels of TR isolates they detected to be comparable to levels of ARAf found in urban air samples in the UK and The Netherlands.

Peanut crops in the United States are unlikely to be a hotspot, as only DMI-susceptible *A. fumigatus* has been isolated from peanut field soil and fresh plant debris [61,92]. However, the latter study showed that the *A. fumigatus* population (including isolates carrying several *cyp51A* mutations) increased significantly in aged compost piles created with waste from the same field where ARAf had earlier been absent. The potato crop setting is considered a likely cold spot in Denmark due to the absence of DMI residues in the harvested tubers and to the fact that waste heaps are avoided for phytosanitary reasons [93].

Fraaije et al. [60] found ARAf in soil from one of three maize fields sampled in Hungary and the Netherlands. Freshly harvested maize samples from continental European locations carried either no *A. fumigatus* or a small population only [94,95]. However, maize soils showed the highest frequency of detection (15%) of resistant isolates in soil samples among the various crop settings sampled in the UK by Tsitsopoulou et al. [83]. In South America, Bedin Denardi et al. [96] showed that 25% of samples from maize crops sampled in Brazil showed itraconazole resistance (but no pan-DMI resistance), and about half of soil samples taken from maize fields in Colombia contained ARAf [97]. Maize silage carried either no *A. fumigatus* [5] or low populations, up to the point at which it was exposed to air, after which the population increased significantly [94,95]. A comparison of maize silage from DMI-treated (double-dose) and untreated plots showed no effect on the DMI-susceptibility of *A. fumigatus* isolates; the single (non-TR) voriconazole-resistant isolate detected was recovered from silage from an untreated plot [95].

There have been a number of investigations of the prevalence of ARAf in the rice setting, again with contrasting findings. Tangwattanachuleeporn et al. [85] detected low rates of incidence in Thailand (6/15 sampled sites with *A. fumigatus*; 2/15 with ARAf). Vaezi et al. [98] found ARAf in ca. 4% of rice paddy soil samples in Iran, and Chowdhary et al. [76] found ARAf in one of five rice paddy areas sampled in India. Chen et al. [99] found ARAf in one of 16 rice soil samples in China at one of five paddy sites sampled (despite the presence of DMI residues in the majority of samples), and Cao et al. [100] recovered ARAf from ca. 5% of 1550 isolates obtained from 886 Chinese paddy fields. In contrast, Duong et al. [40] found a much higher frequency and widespread distribution of ARAf in samples from Vietnam, including samples taken from rice fields—a setting that showed a higher probability of detection of itraconazole-resistant ARAf than uncultivated national parks. However, the correlation with the presence of DMI residues in the rice field soils was weak, and an association could only be concluded on the basis of anecdotal confirmation of DMI use. This was not the case in the study of Cao et al. [100], which isolated ARAf from ca. 6% of samples carrying detectable DMI residues; the highest rate of detection (1/5 samples) was found in the group of soils with the highest overall combined DMI residue.

### 3.5. ARAf in Vegetable, Horticultural and Perennial Crop Settings

Sampling of soil and plant debris in vegetable, horticultural, and perennial crop settings (Supplementary Table S3) has produced a similar diversity of findings to the investigations in arable crops. Among the crop settings investigated at more than one location, only soils taken from Allium, brassica, and tobacco crops failed to yield ARAf, but this is insufficient to characterize them as cold spots.

Grapevine is a crop setting in which DMIs are commonly used, but soils have not consistently yielded ARAf; for example, Riat et al. [101] only found an isolate carrying the G54R mutation in one of 15 vineyard soils sampled in Switzerland, but no pan-DMI resistant isolates at any of the investigated sites. Lago et al. [102] collected isolates of *Aspergillus* spp. (including *A. fumigatus*) from grapevine soils, leaves, and berries in Portugal; while several isolates showed elevated MICs for voriconazole (and to a lesser extent itraconazole), there was no evidence for selection of resistant isolates following multiple applications of the DMI penconazole.

Several studies have highlighted the strawberry crop as a potential hotspot; for example, Chen et al. [99] found a higher frequency of ARAf along with higher DMI residues in strawberry than in other crops analysed in China, and sampling in the US also detected target mutants such as F46Y/M172V/N248T/D255E/E427K in strawberry soil and plant debris, whereas ARAf was absent from other agronomic settings with a history of DMI use [61]. However, strawberry was the only one of five agronomic settings tested in Colombia for which ARAf was not found in soil [97].

Zhou et al. [103] suggested that the greenhouse setting is likely to generate hotspots where high DMI residues coincide with high temperatures under glass, providing a conducive environment for the growth of *A. fumigatus* and the emergence and selection of ARAf. While Ren et al. [64] found ARAf in only ca. 2% of samples from greenhouses, Zhou et al. [103] found it in 80% of samples, although a correlation with DMI residues was lacking.

Several studies involving sampling from organic crops have detected only *cyp51A* wild-type *A. fumigatus*, but this has also been the case for a number of conventional crop settings in which either DMI use has been confirmed, DMI residues have been analyzed for and found, or both (Supplementary Tables S1–S4). Monpierre et al. [104] detected a non-*cyp51A* mutant ARAf in an organic sugar cane field on Martinique but isolated ARAf from only one of 100 samples taken from banana plantations, a crop setting that depends on the intensive use of DMIs. Barber et al. [86] found no differences in the proportion of isolates capable of growing on DMI-amended agar among soil samples collected from organic and DMI-treated apple fields in central Germany. An organic grape field that had received compost from household waste was the single location to yield ARAf in a survey of soils from various agronomic settings in Greece [105]. Soil from olive groves confirmed not to have been treated with DMIs contained the second highest frequency of TR_34_ isolates among the seven agronomic settings sampled in a study in Italy [91].

These findings suggest that there is no clear-cut distinction between the organic and conventional systems in terms of the detection of ARAf. Pan-DMI-resistant isolates have also been isolated from other settings less likely to bring *A. fumigatus* into contact with DMI residues, such as private vegetable gardens [91], and car park soil and tree hollows in Tanzanian hospital grounds [106]; an isolate taken from the grass verge of a European petrol station showed itraconazole resistance [60]. These findings probably reflect the ubiquity of *A. fumigatus* (wild-type and resistant isolates alike) as the result of deposition onto soils and other matrices from widely distributed mixed populations in the air spora. Indeed, Fraaije et al. [60] showed comparable detection rates for pan-azole resistant ARAf in arable crop soils and urban air samples in the Netherlands and the UK. The panmictic nature of the population may also explain the lack of molecular variance in ARAf isolates from farms across central Germany [86], and the absence of gradients in ARAf detection in soils across landscapes such as the Italian peninsula, with its significant regional differences in environmental conditions, cropping patterns, and intensity of DMIs use [91]. A possible trend emerging from the studies reviewed here (Supplementary Tables S1–S4) is that where pan-azole resistant ARAf is found in soils sampled from (growing) crop settings, the widespread TR_34_/L98H and TR_46_/Y121F/T289A mutations tend to be detected, perhaps as the result of deposition from the air spora, whereas greater genetic variation (and higher CFU numbers) are found in waste piles, compost, and soil into which compost has been incorporated.

### 3.6. Further Identification of Hotspots and Coldspots

Our survey of ARAf incidence in various agronomic settings suggests that it is not generally possible to categorize individual crops as hotspots or cold spots merely on the basis of detecting ARAf isolates in soil samples, but rather that the individual settings require more detailed, comparative investigation, such as has been done for flower bulb and cereal cultivation. For other crop settings, it will be important to pinpoint the stages and aspects of the cropping cycle that are most conducive to the amplification of ARAf, with particular reference to the generation and management of plant waste, both in- and off-field.

## 4. The Role of Plant Waste in the Amplification and Spread of ARAf

Several studies have shown that storage of plant-derived waste material can provide a conducive setting both for the growth of *A. fumigatus* in general (e.g., [94]) and for the selection of ARAf in the presence of DMI residues in particular (e.g., [52]). Schoustra et al. [5] detected high *A. fumigatus* (and ARAf) CFUs in flower bulb waste, industrial green waste, and wood chip waste, although sampling of fruit waste yielded no *A. fumigatus* colonies, independent of whether DMI fungicides had been applied ([56]). High CFUs were found in green waste; in a fresh batch (<1 week old), ca. 10% of CFUs were ARAf despite the absence of DMI residues higher than the limit of quantification. Zhang et al. [38] found ARAf in both DMI-free (organic) and DMI-containing compost heaps from flower bulb cultivation, but the proportion of resistant isolates was significantly higher in composting materials carrying DMI residues.

Santoro et al. [94] failed to isolate *A. fumigatus* from a growing maize crop in Italy, whereas Cao et al. [67] showed that it is possible to recover ARAf from DMI-treated tomato plants in the field, demonstrating that the growing crop can harbour resistant genotypes. However, two studies, in particular, have indicated that crop settings may move beyond being mere hosts for *A. fumigatus* or ARAf to become hotspots at the point of plant waste generation. In an investigation of the prevalence of ARAf at different stages of waste management, Hurst et al. [92] found peanut fields soil and postharvest peanut plant debris to be free of *A. fumigatus* (a finding confirmed by Kang et al. [61]), while long-term compost piles of peanut crop waste within the same setting contained a high number of *A. fumigatus* CFUs, including TR_34_/L98H ARAf. Kang et al. [61] found TR_46_-based ARAf in pecan debris from DMI-treated plantations stored at a processing site in the US, but none in soil and plant debris from within the plantations themselves.

Plant waste heaps provide ideal conditions for *A. fumigatus*, which becomes dominant in the microbiota as the temperature rises during the composting process [107]. CFUs in composting materials are considerably higher than those determined for agricultural soils (e.g., [5,86]. Compost supports asexual reproduction in *A. fumigatus*, leading to the mass release of conidia [1,7,8]. Moreover, it exposes the fungus to stress factors (high temperatures, nutritional limitation, hypoxia, and oxidative stress) that may trigger parasexual or sexual reproduction events that are also capable of generating genetic changes [6,12,38,108]. As a result, individual compost heaps can show considerable genetic diversity in terms of mutations in the *cyp51A* gene [94].

High frequencies of ARAf have been detected in urban planted areas, for example, in the air above parks, in botanical gardens and other flowerbed soils, and flowerpot soil from urban locations (Supplementary Table S1). With some exceptions (e.g., [83]), these detection frequencies have also exceeded those for agricultural soils [35,60,84]. Given that DMIs are not applied as intensively in urban settings as in agronomic settings, it is conceivable that plant waste (presumably in the form of compost) from agricultural produce consumed in urban centres creates a local context for the amplification of ARAf, especially if it carries DMI residues (as suggested by Duong et al. [40]), or that previously-composted materials transfer ARAf to urban settings. Schoustra et al. [5] showed that once the breakdown of plant material is complete, the resulting compost is low in *A. fumigatus* CFUs, and some studies have failed to detect ARAf in commercial compost samples [88,94,109]. However, the exclusion of *A. fumigatus* probably depends on how the compost is processed, as both wild-type and ARAf genotypes have been detected in samples of end-product commercial compost in several countries [32,53,84,101]. Shelton et al. [77] tested garden soils from the UK and recovered more CFUs of ARAf (and more *A. fumigatus* CFUs generally) from samples containing compost (either commercial or home-made) than from those that did not. The detection of ARAf in an organic raisin grape field in Greece that had been treated with compost [105] may be an example of the transfer of ARAf into an agronomic setting.

## 5. Approaches to Mitigation

Several authors have referred to the need for antifungal resistance stewardship in all areas of DMI use as a necessary component of a “One Health concept” in order to preserve the availability and efficacy of both the available medical antifungals and agricultural fungicides [24,110,111]. The primary aim of mitigation will be to avoid the mass release of spores of *A. fumigatus*, particularly the spread of ARAf, to limit human exposure [56].

The EUCAST method [112] allows ARAf isolates to be distinguished from the susceptible wild-type based on higher MICs for DMIs in vitro. It has been used to show that individual DMI fungicides vary in their activity towards *A. fumigatus*. Snelders et al. [20] determined MICs for a range of agricultural DMIs (and non-DMI fungicides) against groups of clinical and environmental *A. fumigatus* wild-type and TR_34_/L98H ARAf isolates; they calculated a “correlation effect size” for each fungicide, i.e., a comparison of median MIC values for the respective wild-type and TR_34_/L98H groups. The largest effect size values clustered around itraconazole and five DMI fungicides that are structurally similar to it, showing the greatest similarity in binding mode to a model Cyp51A protein in silico. Jørgensen et al. [21] broadened this approach by determining “MIC elevation factors” (a similar index of the MIC difference between wild-type and resistant isolates) for selected DMI fungicides and different ARAf genotypes. They demonstrated that for DMIs with significant intrinsic activity against the wild-type, the MIC elevation factor varied, depending on the specific ARAf genotype being tested. It follows that different genotypes will be selected and amplified in hotspots by different (combinations of) DMIs, as suggested by Schoustra et al. [5].

For a DMI fungicide that shows intrinsic activity against *A. fumigatus* and a high effect size/MIC elevation factor for a specific ARAf genotype, selection will be strongest if residues of the DMI are within the band of concentrations delineated by the “effect size”. Residue concentrations lower than the wild-type MIC will have a limited impact on the structure of the population. Concentrations higher than the MIC for the ARAf genotype will suppress the entire *A. fumigatus* population. This principle was effectively demonstrated by Jeanvoine et al. [16], who showed that the prevalence of ARAf isolates in sawmill waste rose and then declined again as the concentration of propiconazole present as a residue in the substrate increased.

For DMI fungicides that are highly active against *A. fumigatus*, concentrations of residues present in plant (particularly waste) material need to be brought into relation with the respective MIC for the wild-type. Directly comparing in vitro MICs with residue concentrations in crop substrates is uncertain because the bioavailability of the residues may be limited by the ability of DMIs to adsorb to organic material [58]. Nevertheless, widely-available maximum residue limit values (MRLs) for individual DMI/crop combinations can provide a first indication of potential hotspot settings, particularly if the MRL is directly relevant to the substrate favouring the growth of *A. fumigatus*. Non-active DMIs for which the MRL is lower than the MIC for wild-type *A. fumigatus* are unlikely to select or amplify ARAf genotypes. Changes to the use pattern of highly active DMIs in confirmed hotspots (e.g., reducing the application rate, changing the type and crop stage of application, or bringing forward the timing of the last application) may mitigate selection and amplification by reducing the residue load, particularly at the time of harvest, but this will depend on regulatory approval and demonstration that efficacy against the target pathogen is maintained.

Comparing measured residues (mg/Kg) of specific DMIs in bulb waste piles [52,56] with EUCAST MIC values (mg/L) for *A. fumigatus* wild-type and a selection of ARAf isolates with *cyp51A* mutations [21] shows that the residues can occur in the bulb waste pile hotspot in concentrations that lie within the “MIC band” that separates wild-type and ARAf genotypes. The demonstrable amplification of resistant isolates in bulb waste heaps shows that in this setting at least, residue concentrations can be within the range capable of selecting ARAf genotypes at the expense of the wild-type.

A review of the available studies indicates that an ARAf hotspot typically involves plant waste materials containing DMI residues rather than relating to the fungicidal treatment of the crop per se, suggesting that controlled waste management may provide mitigation for the selective action of DMIs in an otherwise hotspot setting. For crops in which residual organic material is neither processed in the field after harvest (spread directly onto the soil or worked into it) nor directed towards straw, hay, or silage, the drive for a circular economy promotes the eventual return of “waste” organic material to the original setting. However, it comes with the need for appropriate waste management and composting practices that avoid the creation of an ARAf hotspot [12]. This will involve eliminating the long-term accumulation of plant waste in unmanaged piles [56] and prioritizing, where possible, static, undisturbed heaps that are shielded from the outdoor environment, and for which the temperature is allowed to reach 55–60 °C for a sufficiently prolonged period [5,113]. Activities such as screening, shredding, and turning of compost, which trigger the release of significant numbers of conidia [1,3,8,114], will need to be optimized. The hydrolysis of waste at centralized collection sites has also been shown to reduce *A. fumigatus* populations [5,56]. Creating conditions in compost that are unfavourable for the growth of *A. fumigatus* is likely to reduce the probability of resistant genotypes emerging via spontaneous mutations. Research is currently ongoing to test this approach for dealing with the flower bulb waste hotspot in the Netherlands [12]. Finally, further options for waste management may be available that avoid the generation of a hotspot, including the use of residual organic matter for animal feed or as a feedstock for biogas production, alcohol fermentation, or incineration for power generation.

Another research need is to understand the epidemiological implications of the transfer of ARAf beyond the agronomic setting. Flower bulbs have been shown to carry ARAf isolates [53,54,91,115,116,117]. Various fruits and other food and condiment items (along with black tea) are also known to carry *A. fumigatus* conidia [118,119]. While cereal grain was found to be free of ARAf in the Netherlands [5], and resistant genotypes were absent from soil adhering to root vegetables sourced from farm shops and private gardens in the UK [120], other sampling also conducted in the UK (reported by [56]) isolated ARAf from supermarket-bought samples of tea, pepper, onions, and other food commodities originating from around the world. It remains to be determined whether this means that the respective agronomic settings producing these food commodities are hotspots requiring mitigation, whether the rate of detection of ARAf genotypes reflects their prevalence in background populations, or whether they were introduced during postharvest operations along the value chain, including processing, packaging, and transport.

## 6. Concluding Remarks

This literature review indicates that ARAf has been recovered from many of the agronomic settings that have been sampled, but that so far, only those investigations that have focused on the flower bulb and cereal settings have been sufficiently detailed to characterize them respectively as a likely hotspot (in relation to waste stockpiling) and a coldspot. Many of the findings in other crops are restricted to soil sampling, with often low recovery rates for resistant isolates and contradictory findings for the same crop sampled at different sites, both within and between studies and locations. Soil sampling alone is probably of limited value in distinguishing a potential hotspot from the background levels of ARAf isolates that are distributed in, and deposited from, the air spora. Waste management appears to be the most critical determinant of a hotspot, and ongoing studies may provide more detailed insights into particular crop settings to support decision-making for necessary mitigation steps.

## Figures and Tables

**Table 1:** List of requirements for classifying an agronomic setting as a hotspot for the amplification of DMI fungicide resistance in *Aspergillus fumigatus*.

Requirement	Comments
Favourable conditions for growth and multiplication of *A. fumigatus*	Availability of a suitable organic substrate capable of supporting a sizable population of *A. fumigatus*; prevailing conditions of temperature and humidity provide optimal growth conditions, leading to a competitive advantage for *A. fumigatus* within the fungal/microbial community
Exposure of *A. fumigatus* to residual concentrations of DMI fungicides that are selective for resistant genotypes	Residue levels of a specific DMI fungicide exceed its Minimum Inhibitory Concentration (MIC) for wild-type (susceptible) *A. fumigatus*, leading to the selection of resistant *A. fumigatus* genotypes
Mass release of airborne spores of *A. fumigatus* into the environment	Selection of resistant genotypes leads to preferential reproduction and release into the air spora, resulting in an “amplification” of resistance over background levels

## Data Availability

Not applicable.

## Supplementary Materials

The following are available online at https://www.mdpi.com/article/10.3390/microorganisms9122439/s1, Table S1: Findings in connection with the use of DMI fungicides in flower bulb cultivation, Table S2: Findings in connection with the use of DMI fungicides in arable crops, Table S3: Findings in connection with the use of DMI fungicides in vegetable and horticultural crop settings, Table S4: Findings in connection with the use of DMI fungicides in perennial crops. References [121,122,123,124,125,126] are cited in the Supplementary Meterials.
