# Supplementary material for: Selection and Amplification of Fungicide Resistance in Aspergillus fumigatus in Relation to DMI Fungicide Use in Agronomic Settings: Hotspots versus Coldspots
Source: Microorganisms. 2021 Nov 26;9(12):2439. doi: 10.3390/microorganisms9122439 (PMC8704312; doi:10.3390/microorganisms9122439)
Supplement: Supplementary file 1 [file microorganisms-09-02439-s001.zip › microorganisms-1451452-supplementary.pdf]

Table S1. Findings in connection with the use of DMI fungicides in flower bulb cultivation.

Setting	Matrix sampled	*A. fumigatus* detected / ARAf detected [1]	*cyp51A* mutation(s) of ARAf isolates	Confirmation of DMI use / Analysis for DMI residues / Detection of DMI residues[2]	Country[3]	Reference
Flower field	Soil	+ / +	TR_{34}/L98H, TR_{46}/Y121F/T289A	+ / + / +	CO	[42]
	Waste piles / compost	+ / +	TR_{34}/L98H, TR_{34}/L98H/S297T, TR_{46}/Y121F/T289A, TR_{46}/Y121F/M172I/T289A/G448S, TR_{46}/Y121F/T289A/S363P/I364V/G448S, TR^3_{46}/Y121F/M172I/T289A/G448S, TR^4_{46}/Y121F/M172I/T289A/G448S	+ / + / +	NL	[52]
		+ / +	TR_{34}/L98H, TR_{46}/Y121F/T289A, TR_{34}/L98H/F495I, TR_{34}/L98H/S297T/F495I, TR_{92}/Y121F/M172I/T289A/G448S, TR_{46}/Y121F/T289A/I364V	+ / + / +	NL	[5]
		+ / +	TR_{46}/Y121F/T289A, TR_{46}/Y121F/T289A/M172I/G448S TR^3_{46}/Y121F/T289A/M172I/G448S	+ / + / +	NL	[38]
Flowerpots	Soil & compost	+ / +	TR_{34}/L98H	( ) / - / -	FR	[54]
		+ / +	TR_{34}/L98H	+ / - / -	IN	[76]
		+ / +	TR_{34}/L98H, WT	( ) / - / -	IT	[88]
		+ / +	TR_{34}/L98H, TR_{34}/L98H/K240R, TR_{34}/L98H/P443L, TR_{34}/L98H/A460S, TR_{34}/L98H/D481N, TR_{46}/Y121F/T289A, C270R, I242V, WT	( ) / - / -	UK	[77]
Flower beds	Soil	+ / -	-	+ / - / -	BJ	[84]
		+ / +	TR_{34}/L98H	( ) / - / -	CH	[101]
		+ / +	TR_{34}/L98H, TR_{34}/L98H/S297T/F495I, TR_{46}/Y121F/T289A, G448S F46Y/G89G/M172V/N248T/D255E/L358L/E 427K/C454C, G170G, WT	( ) / - / -	CN	[121]
		+ / +	TR_{53}	+ / + / -	CO	[42]
		+ / +	TR_{34}/L98H	( ) / - / -	DK	[109]
		+ / -	-	( ) / - / -	DK	[81]

	+ / +	TR_{34}/L98H	( ) / - / -	IE	[53]
	+ / -	-	( ) / - / -	IN	[76]
	+ / +	TR_{34}/L98H	( ) / - / -	IR	[122]
	+ / -	-	- / - / -	IT	[88]
	+ / +	TR_{34}/L98H	( ) / - / -	IT	[91]
	+ / +	TR_{34}/L98H, TR_{46}/Y121F/T289A, WT	+ /+ / -	MX	[84]
	+ / +	M172V	+ / + / -	NG	[84]
	+ / +	TR_{34}/L98H, WT	( ) / - / -	NL	[32]
	+ / +	TR_{34}/L98H	+ / + / +	PE	[84]
	+ / +	F46Y, M172V, E427K, F46Y, M172V, N248T, D255E, E427K, WT	+ / + / -	PY	[84]
	+ / +	TR_{34}/L98H, TR_{46}/Y121F/T289A	( ) / - / -	TZ	[106]
	+ / +	TR_{34}/L98H, TR_{46}/Y121F/T289A, WT	( ) / - / -	UK	[35]
	+ / +	TR_{34}/L98H	( ) / - / -	UK	[83]
	+ / +	TR_{34}/L98H, TR_{34}/L98H/K240R, TR_{34}^{2}/L98H, TR_{53}, WT	( ) / - / -	UK	[77]

Key:

[1] + detection of an ARAf isolate and/or *A. fumigatus* wild-type isolate in at least one sample of the specified matrix at one or more of the locations selected for sampling.

[2] ( ) data uncertain / not given; +/+/+ DMI use confirmed, DMI residues analysis performed and DMI residues detected; + / + / - DMI residues analysis performed but no DMI residues detected; + / - /- no DMI residues analysis performed.

[3] Country codes according to ISO 3166 (https://www.iso.org/iso-3166-country-codes.html).

WT – wild-type.

Table S2. Findings in connection with the use of DMI fungicides in arable crops

Setting	Matrix sampled	*A. fumigatus* detected / ARAf detected [1]	*cyp51A* mutation(s) of ARAf isolates	Confirmation of DMI use / Analysis for DMI residues / Detection of DMI residues[2]	Country[3]	Reference
Bean	Soil	+ / +	TR_{34}/L98H	( ) / - / -	UK	[83]
Cereals	Soil	+ / +	TR_{34}/L98H, WT	+ / - / -	DE	[86]
		+ / +	TR_{34}/L98H, WT	O / - / -	DE	[86]
		+ / +	F46Y/M172V/E427K	( ) / - -	DE	[60]
		+ / +	TR_{34}/L98H, F46Y/M172V/E427K	+ / - / -	FR	[60]
		+ / +	TR_{34}/L98H	+ / - / -	FR	[88]
		+ / +	TR_{34}/L98H	( ) / - / -	FR	[50]
		+/ -	-	- / + / -	GR	[105]
		+ / -	-	( ) / - / -	HU	[60]
		+ / -	-	+ / - / -	IT	[88]
		+ / +	TR_{34}/L98H, G54E	- / - / -	IT	[91]
		+ / +	TR_{34}/L98H	( ) / - / -	UK	[83]
		+ / -	-	( ) / - / -	UK	[35]
		+ / +	TR_{34}/L98H	+ / - / -	UK	[82]
		+ / +	TR_{34}/L98H, TR_{46}/Y121F/T289A, F46Y/M172V/E427K	+ / - / -	UK	[60]
		+ / +	F46Y/M172V/E427K	- / - / -	UK	[60]
	Fallow soil	+ / +	WT	( ) / - / -	DE	[60]
	Soil and leaves	+ / +	TR_{34}/L98H, WT	+ / - / -	IT	[90]
	Grain	- / -	-	+ / + / +	NL	[5]
	Straw	( ) / -	-	+ / - / -	FR	[88]
	Hay	( ) / -	-	+ / - / -	FR	[88]
	Air sample	+ / +	D100G, V101I, N102H	+ / - / -	UK	[82]
Cotton	Soil	+ / +	TR_{34}/L98H	( ) / - / -	IN	[76]
		+/ -	-	- / + / -	GR	[105]
Maize / Corn	Crop	+ / +	ND	( ) / - / -	BR	[96]
	Soil	+ / +	TR_{46}/Y121F/T289A, WT	- / + / -	CO	[97]
		+ / -	-	( ) / - / -	FR	[50]
		+ / -	-	( ) / - / -	HU	[60]
		+ / +	TR_{34}/L98H	( ) / - / -	NL	[60]
		+ / +	TR_{34}/L98H	( ) / - / -	TH	[85]

		+ / +	TR$_{34}$/L98H, WT	( ) / - / -	UK	[83]
	Silage	+ / -	-	+ / - / -	IT	[95]
		+ / +	WT	- / - / -	IT	[95]
		- / -	-	+ / + / +	NL	[5]
Oilseed rape	Soil	+ / +	TR$_{34}$/L98H	( ) / - / -	UK	[83]
		+ / -	-	+ / - / -	UK	[82]
Mustard	Soil	+ / +	TR$_{34}$/L98H	( ) / - / -	IN	[123]
Pea	Soil	+ / +	TR$_{34}$/L98H	( ) / + / -	CO	[97]
Peanut	Soil	- / -	-	+ / - / -	US	[92]
	Soil + plant debris	+ / -	-	+ / - / -	US	[61]
	Plant debris	- / -	-	+ / - / -	US	[92]
	Plant debris	+ / -	-	+ / - / -	US	[61]
	Long-term compost	+ / +	TR$_{34}$/L98H, F46Y/M172V/N248T/D255E/ E427K, I242V, G254V	+ / - / -	US	[92]
Rice	Soil	+ / +	TR$_{34}$/L98H	+ / + / +	CN	[99]
		+ / +	TR$_{34}$/L98H, TR$_{34}$//L98H/S297T/F495I, TR$_{46}$/Y121F/T289A, TR$_{53}$, F46Y/G89G/M172V/N248T/D255E/L358 L/E247K/C454C	+ / + / +	CN	[100]
		+ / +	TR$_{34}$/L98H	+ / - / -	IN	[76]
		+ / +	TR$_{34}$/L98H, WT	+ / - / -	IR	[98]
		+ / +	TR$_{34}$/L98H	+ / - / -	TH	[85]
		+ / +	TR$_{34}$/L98H	( ) / + / +	VN	[40]
Soybean	Soil	( ) / -	-	( ) / - / -	TH	[85]
Sugar beet	Soil	+ / +	TR$_{34}$/L98H	( ) / - / -	BE	[60]
	Fallow soil	+ / -	-	( ) / - / -	NL	[60]
Sunflower	Soil	+/ -	-	- / + / -	GR	[105]
		+ / -	-	( ) / - / -	HU	[60]
		- / -	-	( ) / - / -	IT	[91]
	Soil + plant debris	+ / +	TR$_{34}$/L98H, G54E	- / - / -	RO	[124]

Key:.

[1] + detection of an ARAf isolate and/or *A. fumigatus* wild-type isolate in at least one sample of the specified matrix at one or more of the locations selected for sampling.

[2] ( ) data uncertain / not given; +/+/+ DMI use confirmed, DMI residues analysis performed and DMI residues detected; + / + / - DMI residues analysis performed but no DMI residues detected; + / - /- no DMI residues analysis performed.

[3] Country codes according to ISO 3166 (https://www.iso.org/iso-3166-country-codes.html).

O = Organic.

WT – wild-type.

ND – not determined.

Table S3. Findings in connection with the use of DMI fungicides in vegetable and horticultural crop settings

Setting	Matrix sampled	*A. fumigatus* detected / ARAf detected [1]	*cyp51A* mutation(s) of ARAf isolates	Confirmation of DMI use / Analysis for DMI residues / Detection of DMI residues[2]	Country[3]	Reference
Alfalfa	Soil	- / -	-	( ) / - / -	IT	[91]
Alliums (onion, garlic, shallot)	Soil	+ / -	-	( ) / - / -	CN[b]	[64]
		+ / -	-	+ / + / ( )	CN	[99]
	Bulb	( ) / -	-	( ) / - / -	JP	[116]
Aubergine / Eggplant	Soil	+ / -	-	+ / + / ( )	CN	[99]
		+ / -	-	( ) / - / -	CN[b]	[64]
		+ / +	TR$_{34}$/L98H	( ) / - / -	IN	[123]
Beetroot	Soil ex-field[a]	+ / -	-	( ) / - / -	UK	[120]
Brassica	Soil	+ / -	-	+ / + / ( )	CN	[99]
	Soil & plant debris	+/ -	-	O / - / -	US	[61]
Carrot	Soil	+ / -	-	( ) / - / -	CN[b]	[64]
		+ / +	TR$_{46}$/Y121F/T289A, WT	- / + / -	CO	[97]
	Soil ex-field[a]	+ / -	-	( ) / - / -	UK	[120]
Caraway	Soil	+ / -	-	( ) / - / -	CN[b]	[64]
Cassava	Soil	+ / +	TR$_{34}$/L98H	( ) / - / -	TH	[85]
Chili	Soil	+ / +	TR$_{34}$/L98H	( ) / - / -	IN	[123]
		+ / +	-	( ) / - / -	IN	[76]
Clover	Soil	+/ -	-	- / + / -	GR	[105]
Colocasia	Root	( ) / -	-	( ) / - / -	JP	[116]
		+ / -	-	( ) / - / -	UK	[83]
Coriander	Soil	+ / +	TR$_{34}$/L98H, ~~TR$_{34}$~~ TR$_{34}$/L98H/S297T/F495I, TR$_{46}$/Y121F/T289A	+ / + / +	CN[b]	[103]
		+ / -	-	( ) / - / -	CN[b]	[64]
Cowpea	Soil	+ / -	-	( ) / - / -	CN[b]	[64]
Cucurbit (calabash, cucumber, Luffa sp., pumpkin, watermelon, white gourd)	Soil	+ / +	TR$_{34}$/L98H	+ / + / +	CN[b]	[103]
		+ / -	-	( ) / - / -	CN[b]	[64]
		+ / +	TR$_{46}$/Y121F/T289A	( ) / - / -	CN[b]	[64]
		+ / -	-	+ / + / ( )	CN	[99]
		+ / +	TR$_{34}$/L98H, F46Y, M172V, N248T, D255E	+ / - / -	IT	[88]

	Soil & plant debris	+/ -	-	O / - / -	US	[61]
		+/ -	-	+ / - / -	US	[61]
	Air	+ / -	-	+ / - / -	JP	[125]
Edible burdock	Root	( ) / -	-	( ) / - / -	JP	[116]
Fennel	Soil	+ / +	TR_{34}/L98H, WT	+ / + / +	CN[b]	[103]
Fenugreek	Soil	+ / +	TR_{34}/L98H, TR_{46}/Y121F/T289A	( ) / - / -	IN	[123]
Lavender	Soil	+ / -	-	( ) / - / -	UK	[35]
Lettuce	Soil	+ / +	TR_{34} /L98H/S297T/F495I, WT	+ / + / +	CN[b]	[103]
Jerusalem artichoke	Soil ex-field[a]	+ / -	-	( ) / - / -	UK	[120]
Parsnip	Soil ex-field[a]	+ / -	-	( ) / - / -	UK	[120]
Pea	Soil	+ / +	TR_{34}/L98H,WT	+ / + / +	CN[b]	[103]
Pepper	Soil	+ / +	TR_{46}/Y121F/T289A, TR_{34}/L98H/S297T/F495I	+ / + / +	CN	[99]
		+ / -	-	( ) / - / -	CN[b]	[64]
Potato	Soil	+ / +	TR_{46}/Y121F/T289A, $TR_{53,}$ WT	- / + / -	CO	[97]
		+ / +	TR_{34}/L98H	( ) / - / -	UK	[83]
		+ / +	TR_{34}/L98H, TR_{46}/Y121F/T289A	( ) / - / -	IN	[123]
		+ / +	TR_{34}/L98H, G45E	( ) / - / -	RO	[124]
	Soil ex-field[a]	+ / -	-	( ) / - / -	UK	[120]
Rape	Soil	+ / -	-	( ) / - / -	CN[b]	[64]
Rose	Soil	+ / +	TR_{34}/L98H	( ) / - / -	IN	[123]
Strawberry	Soil	( ) / -	-	( ) / + / -	CO	[97]
		+ / +	TR_{34}/L98H; TR_{34}/L98H/S297T/F495I; TR_{46}/Y121F/T289A, WT	+ / + / +	CN	[99]
		+ / +	TR_{34}/L98H/S297T/F495I, TR_{46}/Y121F/T289A	( ) / -/ -	CN	[64]
	Soil & plant debris	+ / +	Y46F/V172M/T248N/E255D/K427E	+ / - / -	US	[61]
Tobacco	Soil	- / -	-	( ) / - / -	IT	[91]
		+/ -	-	- / + / -	GR	[105]
Tomato	Crop	+ / +	TR_{34}/L98H, WT	+ / - / -	CN	[67]
	Soil	+ / +	TR_{34}/L98H/S297T/F495I, WT	+ / + / +	CN	[67]
		+ / -	-	+ / + / ( )	CN	[99]
		+ / -	-	( ) / - / -	CN[b]	[64]
		- / -	-	( ) / - / -	IT	[94]

	Soil & plant debris	+/ -	-	+ / - / -	US	[61]
Water spinach	Soil	+ / -	-	+ / + / ( )	CN	[99]
Vegetable fields	Soil	+ / -	-	( ) / - / -	CH	[101]
		+ / +	TR_{34}/L98H, TR_{46}/Y121F/T289A, WT	+ / + / +	FR	[126]
		+ / -	-	O / + / -	FR	[126]
		+/ -	-	- / + / -	GR	[105]
		+ / +	TR_{34}/L98H	- / - / -	IT	[91]
		+ / +	TR_{34}/L98H, G54R	- / - / -	TH	[85]
	Soil & Plant debris	+ / +	TR_{34}/L98H, WT	+ / - / -	IT	[90]

Key:

1 + detection of an ARAf isolate and/or A. fumigatus wild-type isolate in at least one sample of the specified matrix at one or more of the locations selected for sampling

2 ( ) data uncertain / not given; +/+/+ DMI use confirmed, DMI residues analysis performed and DMI residues detected; + / + / - DMI residues analysis performed but no DMI residues detected; + / - /- no DMI residues analysis performed

3 Country codes according to ISO 3166 (https://www.iso.org/iso-3166-country-codes.html)

a Soil adhering to root vegetable

b Greenhouse crops

O = Organic

WT – wild-type

Table S4. Findings in connection with the use of DMI fungicides in perennial crops

Setting	Matrix sampled	*A. fumigatus* detected / ARAf detected [1]	*cyp51A* mutation(s) of ARAf isolates	Confirmation of DMI use / Analysis for DMI residues / Detection of DMI residues[2]	Country[3]	Reference
Apple	Soil	+ / +	TR_{34}/L98H, WT	+ / - / -	DE	[86]
Apple	Soil	+ / +	TR_{34}/L98H, WT	O / - / -	DE	[86]
Apple	Soil	+ / +	TR_{34}/L98H	+ / - / -	IT	[88]
Apple	Soil	+ / +	TR_{34}/L98H	+ / - / -	IT	[91]
Apple	Soil	+ / +	TR_{34}/L98H	- / - / -	IT	[91]
Apple	Soil	+ / -	-	+ / + / -	FR	[126]
Apple	Soil	+/ -	-	- / + / -	GR	[105]
Apple	Soil & plant debris	+/ -	-	+ / - / -	US	[61]
Banana	Soil	+ / +	WT	+ / - / -	MQ	[104]
Blueberry	Soil & plant debris	+/ -	-	+ / - / -	US	[61]
Cherry	Soil	+/ -	-	- / + / -	GR	[105]
Citrus	Soil	+ / +	TR_{34}/L98H	( ) / - / -	IT	[91]
Citrus	Soil	+ / -	-	+ / + / -	CN	[99]
Citrus	Soil	+/ -	-	- / + / -	GR	[105]
Citrus	Soil & leaves	+ / -	-	+ / - / -	IT	[90]
Citrus	Soil & plant debris	+/ -	-	+ / - / -	US	[61]
Grapevine	Soil	+ / -	-	( ) / - / -	CN[b]	[64]
Grapevine	Soil	+ / +	G45R	( ) / - / -	CH	[101]
Grapevine	Soil	+ / -	-	( ) / + / -	GR	[105]
Grapevine	Soil	+ / +	TR_{46}/Y121F/T289A	O / + / -	GR	[105]
Grapevine	Soil	- / -	-	+ / - / -	IT	[88]
Grapevine	Soil	- / -	-	( ) / - / -	IT	[94]
Grapevine	Soil	+ / +	TR_{34}/L98H	+ / - / -	IT	[91]
Grapevine	Soil	+ / +	G54E	- / - /-	IT	[91]
Grapevine	Soil & plant debris	+/ -	-	+ / - / -	US	[61]
Grapevine	Soil, leaves, berries	+ / +	ND	+ / - / -	PT	[102]
Grapevine	Soil, leaves, berries	+ / +	ND	O / - / -	PT	[102]
Grass	Soil	+ / -	-	( ) / - / -	UK	[60]

		+ / +	TR_{34}/L98H	( ) / - / -	UK	[83]
Oil palm	Soil	( ) / -	-	( ) / - / -	TH	[85]
Olive	Soil	+/ -	-	- / + / -	GR	[105]
		+ / +	TR_{34}/L98H	- / - / -	IT	[91]
	Soil & leaves	+ / -	-	+ / - / -	IT	[90]
Peach	Soil	+/ -	-	- / + / -	GR	[105]
Pecan	Soil & plant debris	+ / -	-	+ / - / -	US	[61]
	Plant Debris[a]	+ / +	TR_{46}/Y121F/T289A	+ / - / -	US	[61]
Sugar cane	Soil	+ / +	WT	O / - / -	MQ	[104]
		+ / -	-	- / - / -	TH	[85]
Tea	Soil	+ / -	-	+ / + / -	CN	[99]
		+ / +	TR_{34}/L98H	+ / - / -	IN	[76]

Key:

1 + detection of an ARAf isolate and/or A. fumigatus wild-type isolate in at least one sample of the specified matrix at one or more of the locations selected for sampling

2 ( ) data uncertain / not given; +/+/+ DMI use confirmed, DMI residues analysis performed and DMI residues detected; + / + / - DMI residues analysis performed but no DMI residues detected; + / - /- no DMI residues analysis performed

3 Country codes according to ISO 3166 (https://www.iso.org/iso-3166-country-codes.html)

O = Organic

WT – wild-type

ND – not determined.

a includes isolation of pan-DMI resistant genotype from pecan waste piles at processing sites

b Greenhouse crop
